# An immune-related model based on INHBA, JAG2 and CCL19 to predict the prognoses of colon cancer patients

**DOI:** 10.1186/s12935-021-02000-z

**Published:** 2021-06-08

**Authors:** Xuankun Yang, Jia Yan, Yahui Jiang, Yaxu Wang

**Affiliations:** 1 Department of Gastrointestinal Surgery, The Second Affiliated Hospital of Chongqing Medical University, No. 288 Tianwen Road, Nanan District, Chongqing, 401336 China; 2 Department of General Surgery, Hechuan District People’s Hospital, Chongqing, China; 3 Department of Gastroenterology, University-Town Hospital of Chongqing Medical University, Chongqing, China

**Keywords:** CCa, Immune-related genes, Immune-related risk score, Prognosis

## Abstract

**Background:**

Colorectal cancer (CRC) is the leading cause of cancer deaths and most common malignant tumors worldwide. Immune-related genes (IRGs) can predict prognoses of patients and the effects of immunotherapy. A series of colon cancer (CCa) samples from The Cancer Genome Atlas (TCGA) were analyzed to provide a new perspective into this field.

**Methods:**

Differential IRGs and IRGs with significant clinical outcomes (sIRGs) were calculated by the limma algorithm and univariate COX regression analysis. The potential molecular mechanisms of IRGs were detected by PPI, KEGG and GO analysis. Immune-related risk score model (IRRSM) was established based on multivariate COX regression analysis. Based on the median risk score of IRRSM, the high-risk group and low-risk group were distinguished. The expression levels of IHNBA and JAG2 and relationships between IHNBA and clinical features were verified by RT-qPCR.

**Results:**

6 differential sIRGs of patients with CCa were selected by univariate COX regression analysis. Based on the sIRGs (INHBA, JAG2 and CCL19), the IRRSM was established to predict survival probability of CCa patients and to explore the potential correlations with clinical features. Furthermore, IRRSM reflected the infiltration status of 22 types of immune cells. The expression levels of IHNBA and JAG2 were higher in CCa tissues than that in adjacent normal tissues. The expression levels of IHNBA and JAG2 were increased in advanced T stages.

**Conclusion:**

Our results illustrated that some sIRGs showed the latent value of predicting the prognoses of CCa patients and the clinical features. This study could provide a new insight for immune research and treatment strategies in CCa patients.

**Supplementary Information:**

The online version contains supplementary material available at 10.1186/s12935-021-02000-z.

## Background

With approximate 1.8 million new cases and 0.8 million deaths per year, colorectal cancer (CRC) is the 3rd most common malignant tumors and the 2nd leading cause of cancer deaths worldwide [1–3]. Despite the significances of surgery combining with adjuvant therapies including chemotherapy and radiotherapy have been highlighted, and the progresses in the increasing replacement treatments such as immunotherapy also light the path for patients with CRC, the limited response rate, heterogeneous tolerance and the resulting local recurrence and distant metastasis pose severe challenges to the survival of CRC patients, with 5-year postoperative survival rate for only 27% [4, 5]. Therefore, growing numbers of researchers have been focusing on explore some novel and promising predicting methods and attempting to beforehand distinguish patients with considerable sensibility, thus increase the treatment efficacy from a new perspective.

With the more insightful recognition of the importance of tumor immunological characteristics and functions such as the recruitment and infiltration of immune cells on modulating immune responses [6, 7], emerging immune-related molecules and regulators including PD-1 and PD-L1 have been identified and demonstrated to be closely related to the effectiveness of immunotherapy [8, 9]. Zhao et al. established a personalized prognostic signature based on 68 immune genes to achieve accurate prognostic stratification for patients with high-grade serous ovarian cancer [10]. The overexpression of FAM83H was verified to predict the worse prognosis of pancreatic cancer [11].

Consisting of immune cells, abundant immune molecules and other regulating cytokines, tumor microenvironment (TME) closely associating with the tumor immune response processes, is the crucial concern of discovering immune-related markers [12, 13]. In the past dozens, immune-related genes (IRGs) have been demonstrated to play crucial roles in the occurrence, development and prognosis of various tumors [14, 15]. The IRGs biomarkers in immune microenvironment (IME) also exist potential for predicting the sensitivity of immunotherapy [16–18]. However, there are real differences among the IRGs identified and assessed in various CRC-related researches, besides, the value of IRGs in the IME for prognosis assessments of CRC remain problematic and needed further verifications by large-scale prospective studies and amounts of basic experiments.

Therefore, we designed this study to further investigate the clinical values of IME and IRGs in evaluating the prognoses of patients with CRC. We extracted the IRGs which associated with CRC from IME, combining with their clinicopathological features, and further evaluate the relationships between IRGs and overall survival (OS). We attempted to develop an immune-related risk score model (IRRSM) based on the related IRGs to predict the prognosis of patients with CRC. The results of this study shed light on the underlying mechanisms of IRGs in the progression of CRC, and the establishment of the appropriate and accurate model will provide a new perspective for clinical decision-making.

## Methods

### Human colorectal cell lines and clinical tissues

Colon cancer (CCa) tissues and adjacent normal tissues were collected from 56 patients admitted to The Second Affiliated Hospital of Chongqing Medical University and diagnosed with CCa. Human colonic epithelial cell line (NCM460) and CCa cell lines (CACO-2, HT29, SW480 and HCT116) were purchased from the American Type Culture Collection (Manassas, Virginia, USA). Cells were cultured in RPMI 1640 and DMEM supplemented with 10% fetal bovine serum (FBS), 100 U/ml penicillin and 100 mg/ml streptomycin (Gibco, Gaithersburg, MD, USA). Cells were incubated at 37 °C in 5% CO_2_.

### Data download and preprocess and the analysis of differential genes and differential IRGs

A series of transcriptome RNA-sequencing data of 39 normal colon samples and 398 CCa samples were downloaded from the TCGA data portal (https://portal.gdc.cancer.gov/). Clinical data of these patients were downloaded and extracted (the patients with OS ≤ 30 days were excluded because these patients probably died of some unpredictable factors such as hemorrhage and infection). These data were updated on May 7, 2020. RNA-seq results were combined into a matrix file using a merge script of the Perl language (http://www.perl.org/). The Ensembl database (http://asia.ensembl.org/index.html) was used to convert the Ensembl ID of genes into a matrix of gene symbols. IRGs participating in the immune activity were screened from the Molecular Signatures Database v4.0 (Immune system process M13664, Immune response M19817, http://www.broadinstitute.org/gsea/msigdb/index.jsp).

The limma package (https://bioconductor.org/packages/release/bioc/html/limma.html) of R software was used to screen differential genes in colon tumor and adjacent non-tumor tissues. We defined analysis data of differential genes with the screening value of “FDR < 0.05, log2| FC |> 1 and P < 0.05”. The differential IRGs were extracted from the differential genes. In order to explore the interactions of these genes, a PPI network of these genes was constructed by the STRING online database (https://string-db.org/). PPI networks can show relationships of many interacting genes. The standard of a core gene is no less than five node degrees. CytoHubba of Cytoscape software version 3.7.2 was used to demonstrate PPI results. Functional enrichment analysis was performed to explore the underlying molecular mechanisms of differential IRGs through the Gene Ontology (GO) and Kyoto Encyclopedia of Genes and Genomes (KEGG) pathways. GO and KEGG pathways were based on cluster profiler, org.Hs.eg.db, and enrichplot packages of R software.

Differential IRGs with significant clinical outcomes in CRC patients were confirmed as sIRGs. Univariate COX regression analysis was used to select sIRGs (*P* < 0.05). The protective and deleterious parts of sIRGs were selected based on Hazard ratio (HR). These sIRGs were prepared for the subsequent study.

### Establishment of the immune-related risk score model (IRRSM)

In order to identify the reliability, sIRGs were analyzed by the multivariate COX regression analysis. The screened sIRGs were used to establish the IRRSM. To detect the clinical prognostic outcomes, we created an IRRSM to divide CCa patients into the high-risk group and the low-risk group by the median risk score. IRRSM was established through the expression data multiplied by Cox regression coefficients. The formula was shown as followed, [Expression levels of INHBA * (0.053340)] + [Expression levels of JAG2 * (0.039053)] + [Expression levels of CCL19 * (0.044074)]. The value of IRRSM was used to assess various subtypes of CCa patients. The CIBERSORT (http://cibersortx.stanford.edu/) database was employed to evaluate the tumor infiltrating immunocytes. The immune-related scores of various genes were included in the CIBERSORT database. So, we scored according to the amounts of immune-related genes carried by each sample, and then get the immune score of each sample. The immune infiltration levels in CCa patients were downloaded, and the correlations between IRRSM and immune cells infiltration were detected.

In order to verify the relationships between the sIRGs and clinical characteristics of CCa patients, we analyzed the correlations between the IRRSM and clinical features, of which the “the staging method of TNM” is the most conventional way to describe the tumor status. The CCa “T-stage” division is on the basis of the extent and depth of tumor invasion, with a lighter and less extensive invasive status in the early T-stages. “N-stage” reflects the conditions of lymph node metastasis with less and smaller metastatic lymph nodes in the early N-stages. “M-stage” is distinguished on the basis of whether the tumor exists distant metastasis, and poor tumor conditions usually associated with advanced M-stages. Additionally, “stage” is a complex staging method, and it always combines with T-stages, N-stages and M-stages to separate CCa patients into I, II, III and IV stage.

### Real-time quantitative PCR

Based on the manufacturer's instructions, total RNAs of CCa tissues, colon normal tissues, colon cell lines and colon epithelial cell line were extracted by TRIzol (Invitrogen). RNA (1 μg) and PrimeScript RT kit (Osaka, Japan of TaKaRa) were used to get reversed transcribe cDNA. Based on the SYBR-Green method (TaKaRa), quantitative PCR was detected by an ABI 7500 real-time PCR system (Applied Biosystems). The reaction cycle conditions were 95 °C for 30 s, followed by 40 cycles of 95 °C for 5 s and 60 °C for 34 s; the primer sequences were shown in Table 1. The measurements of each cDNA samples were replicated three times.

**Table 1:** The primer sequences of JAG2, INHBA and β-actin

JAG2	F primer (5ʹ-3ʹ)	CCCTCCTCGTGAAAGTGCAT
	R primer (5ʹ-3ʹ)	ATACAAAAGGGACAGCACCGAA
INHBA	F primer (5ʹ-3ʹ)	ACAGGCACTTTCCTACCCAA
	R primer (5ʹ-3ʹ)	GCACACGATTGTTCTTTTACCAGT
β-actin	F primer (5ʹ-3ʹ)	AAACGTGCTGCTGACCGAG
	R primer (5ʹ-3ʹ)	TAGCACAGCCTGGATAGCAAC

*F primer* forward primer, *R primer* reverse primer

### Statistical analysis

In order to verify the prognoses of CCa patients, the ROC curve was drawn by the survival ROC package of the R software. We drew a nomogram plot to forecast the survival probabilities of CCa patients using the rms package of R software. Principal component analysis (PCA) was used to illustrate the expression of CCa samples. Univariate Cox regression analysis, Pearson correlation analysis and multivariate regression analysis were utilized to confirm the sIRGs. Kaplan‐Meier curve was used to estimate the OS of high‐risk group and low‐risk group and to verify the independent prognostic factors of CCa patients. Radargram was drawn by the limma package of the R software. All statistical analysis was conducted using SPSS21.0 software (SPSS Inc, Chicago, IL) and GraphPad Prism5 (GraphPad Software Inc, La Jolla, CA). Variations in clinical parameters were determined using independent t-tests. *P* < 0.05 was considered statistically significant. The error bar indicated SD, and all experiments were conducted for 3 times.

## Results

### Differential expression of mRNAs and IRGs

**Fig. 1:**
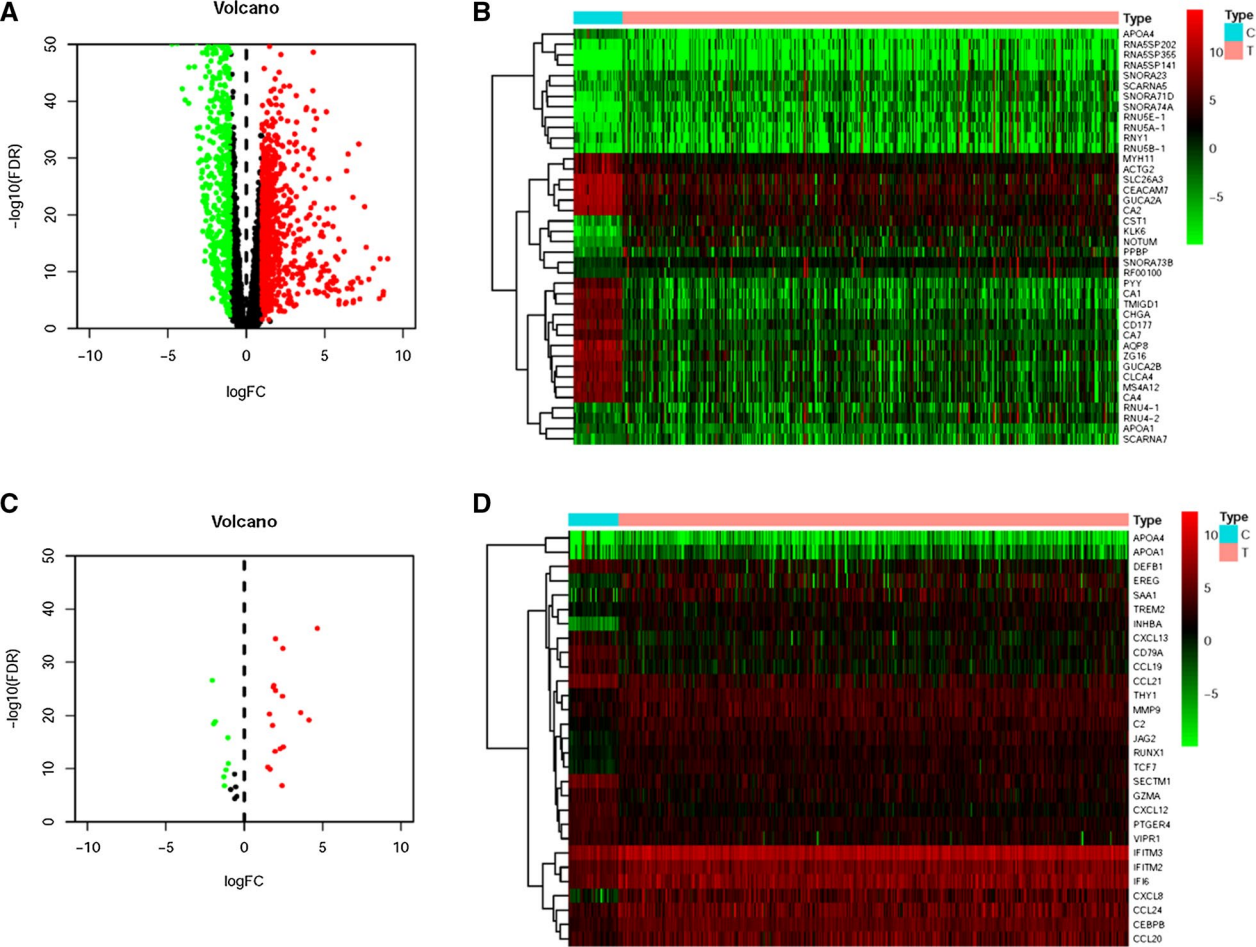
Differentially expressed CCa genes and immune-related genes. Volcano plot (**A**) and Heatmap (**B**) illustrated the differentially expressed genes between CCa tissues and adjacent non-tumor tissues. Immune-related genes (IRGs) with different expression levels were showed in the volcano plot (**C**) and heatmap (**D**). FDR < 0.05, log_2_ | FC |> 1 and P < 0.05

Transcriptome data and clinical data of patients with CCa were acquired from TCGA database. Next, transcriptome data were processed to convert the data ensembl ID into gene names. Based on limma algorithm, we screened 1550 differentially expressed CCa genes, of which 667 were down-regulated and 883 were up-regulated (Fig. 1A). Next, the 20 most up-regulated and down-regulated genes were respectively confirmed by the values of log2∣FC∣and the heatmap was illustrated in the Fig. 1B. From the immune system process M13664 and immune response M19817 of Molecular Signatures Database, we further identified 331 IRGs, of which 29 genes including 12 down-regulated and 17 up-regulated IRGs were recognized to be associated with CCa through the correlation analysis (Additional file 1: Figure S1) (Fig. 1C, D).

### The characteristics of IRGs

**Fig. 2:**
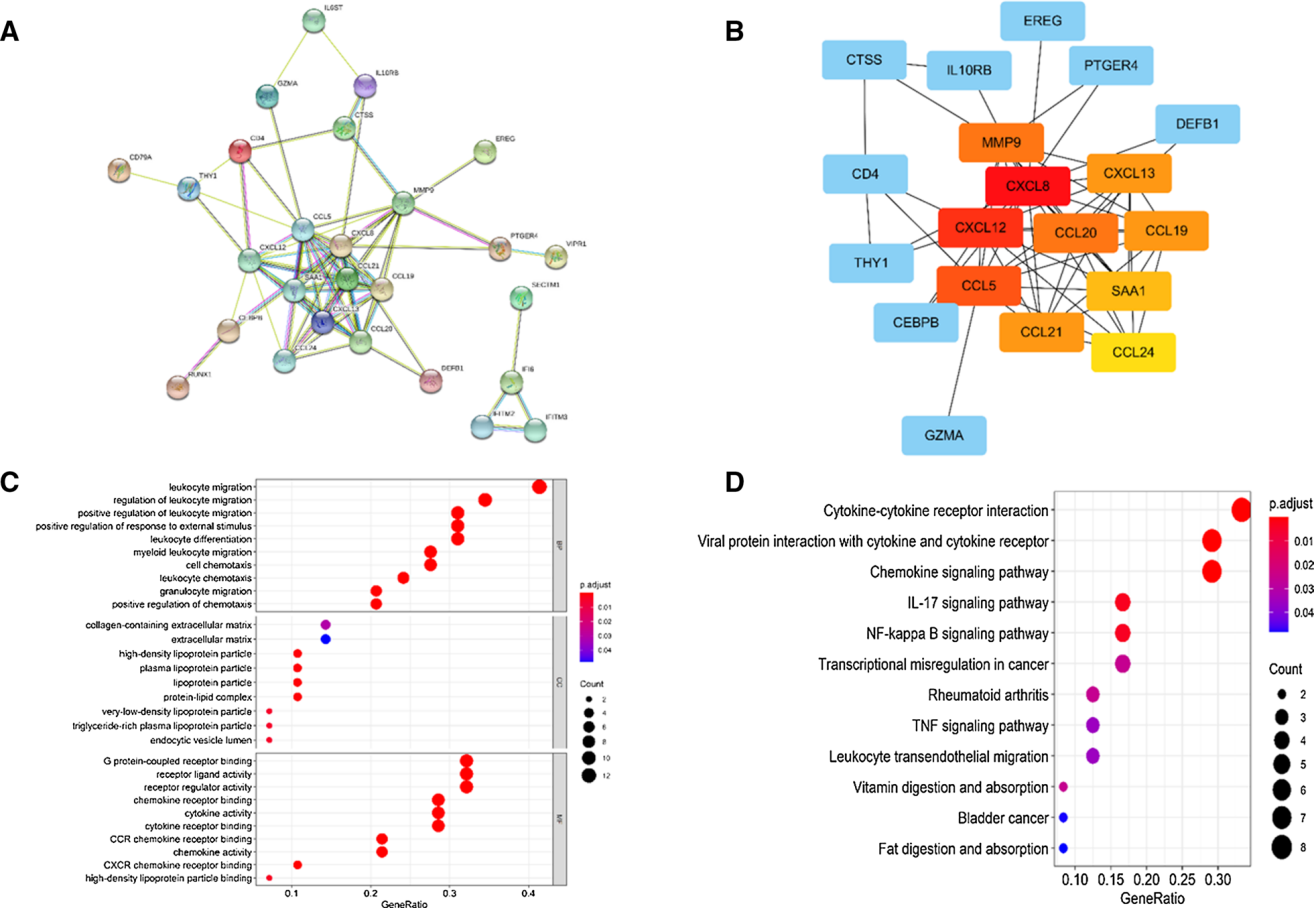
The functional enrichment analysis of differentially expressed IRGs. PPI network (**A**) of IRGs and the core IRGs (**B**). The top pathways of IRGs were shown in biological process (BP), cellular component (CC), molecular function (MF) (**C**), and top KEGG pathways (**D**)

To explore the underlying regulating relationships among IRGs, we detected the interactions of these IRGs by protein–protein interaction (PPI) network analysis (Fig. 2A). We defined that the core gene had no less than five node degrees. Following this criterion, CXCL8, CXCL12, SAA1, CCL20, CCL5, CCL19, MMP9, CXCL13, CCL21 and CCL24 were regarded as the core genes among the 29 IRGs (Fig. 2B). Furthermore, functional enrichment analysis was performed to explore the molecular mechanisms of IRGs through the GO and KEGG. As illustrated in the Fig. 2C, “leukocyte migration”, “extracellular matrix and collagen-containing extracellular matrix” and “G protein-coupled receptor binding, receptor ligand activity and receptor regulator activity” were the most enriched terms in biological processes (BP), cellular components (CC) and molecular functions (MF), respectively. “Cytokine-cytokine receptor interaction” was identified to be the most enriched among the KEGG pathways of IRGs (Fig. 2D).

### The relevancies of IRGs and prognosis of CCa patients

To further explore the correlations between IRGs and clinical outcomes, we employed univariate COX regression analysis and identified 6 sIRGs, such as CXCL12, INHBA, RUNX1, JAG2, CCL19 and IFITM2. As illustrated in the Table 2, 6 sIRGs were the deleterious genes.

**Table 2:** The results of univariate Cox regression

Gene	HR	HR.95% low	HR.95% high	*P* value
CXCL12	1.076477	1.006418	1.151414	0.031848
INHBA	1.053879	1.020953	1.087867	0.001193
RUNX1	1.076588	1.013037	1.144125	0.017443
JAG2	1.030391	1.010569	1.050601	0.002520
CCL19	1.042021	1.020599	1.063892	0.000102
IFITM2	1.002334	1.000108	1.004566	0.039864

*HR* Hazard ratio

### Clinical outcomes of the high-risk group and the low-risk group

**Fig. 3:**
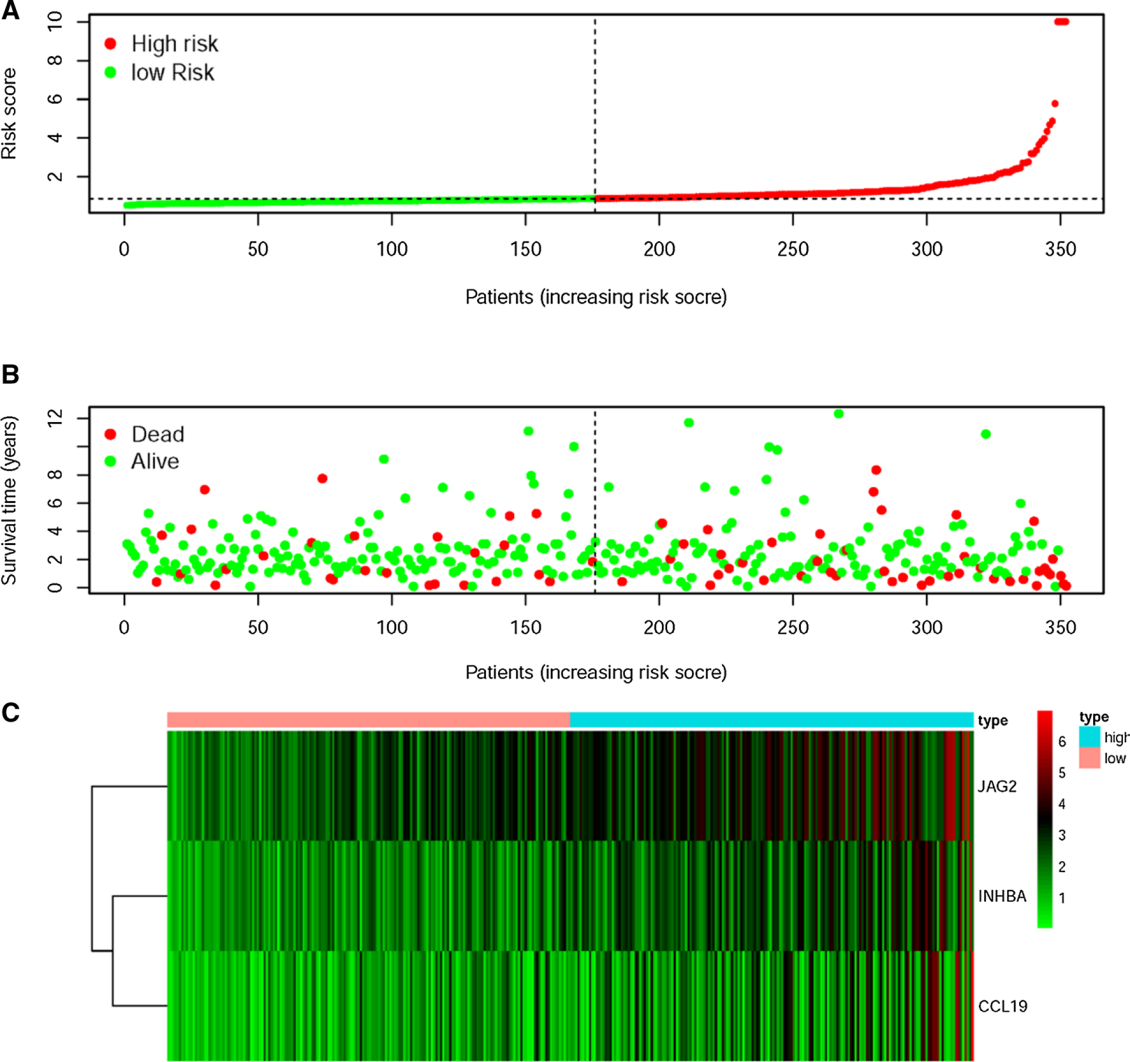
Immune-related risk score model (IRRSM) was created by sIRGs. The distribution of high-risk group and low-risk group (**A**). Survival status of the high-risk and the low-risk group (**B**). The heatmap of the expression levels of three sIRGs (**C**)

**Fig. 4:**
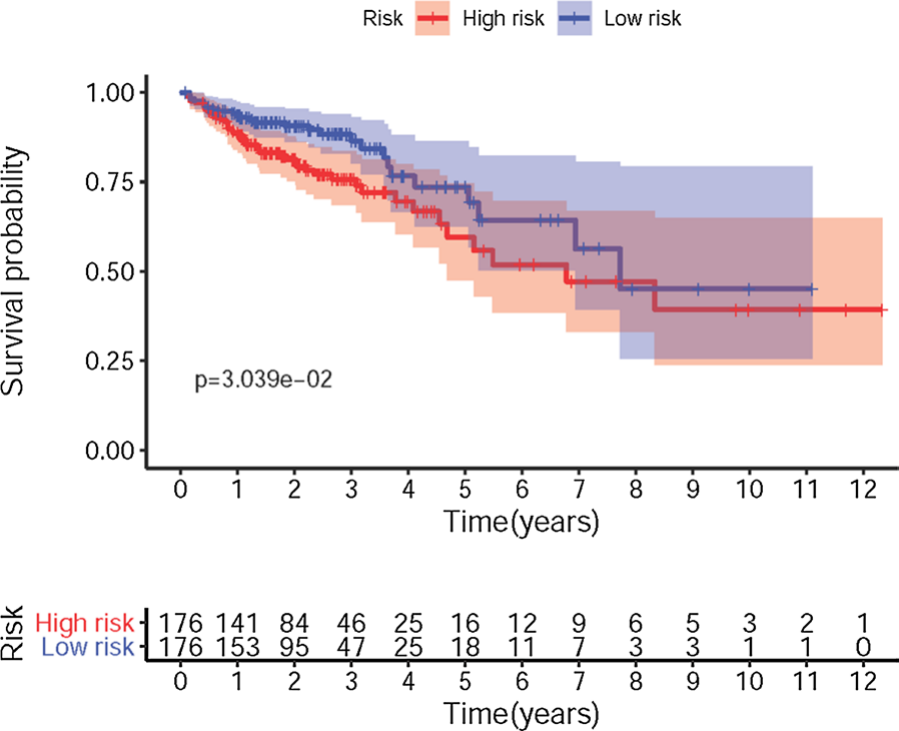
Survival curve of the high-risk group and low-risk group. The Kaplan‐Meier survival curve of the low-risk group and the high-risk group

In order to establish the IRRSM, we selected the 3 sIRGs (INHBA, JAG2 and CCL19) among the 6 sIRGs using multivariate COX regression analysis (Table 3). According to the IRRSM, the CCa samples were divided into the high-risk group and the low-risk group (Fig. 3A). The mortalities of patients with the higher risk scores were significantly higher than whom with the lower risk scores (Fig. 3B). With the increase of risk scores, the expression levels of CCL19, INHBA and JAG2 were enhanced (Fig. 3C). Besides, in the IRRSM, the survival probability of the low-risk group was significantly higher than that of the high-risk group (Fig. 4).

**Table 3:** The results of multivariate Cox regression

Gene	Coefficients	HR	HR.95% low	HR.95% high	*P* value
CCL19	0.044074	1.045060	1.023817	1.066744	2.59E−05
INHBA	0.053340	1.054788	1.019940	1.090826	0.001859
JAG2	0.039053	1.039825	1.020505	1.059511	4.48E−05

*HR* Hazard ratio

### The relationships between IRRSM and clinical characteristics

**Fig. 5:**
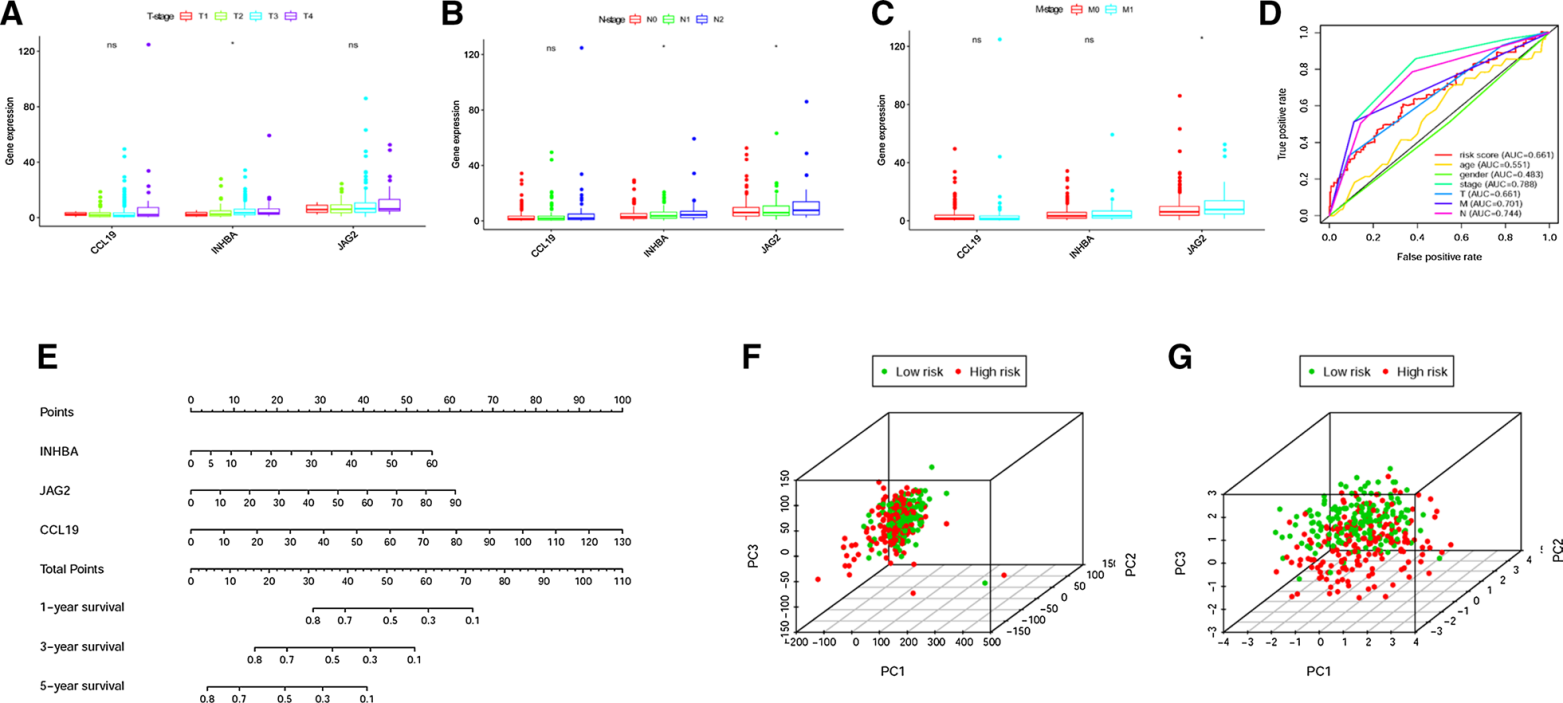
The relationships between the sIRGs and clinical features and the analysis of immune status of the high-risk group and low-risk group by PCA. Relationships between sIRGs and clinical characteristics. The expression levels of INHBA were increased in more advanced T-stages. **A** The expression levels of INHBA and JAG2 were increased in advanced N-stages. **B** The expression levels of JAG2 were decreased in early M-stages. **C** (*P < 0.05; **P < 0.01; ***P < 0.001) The ROC curves of various clinical features and IRRSM. **D** Nomogram was used for predicting 1, 3, and 5-year survival probability of CCa patients. **E** The high‐risk group and the low‐risk group were analyzed by the whole gene sets (**F**). The high‐risk group and the low‐risk group were analyzed based on the immune risk gene sets (**G**)

To further understand the underlying relationships between clinical characteristics of CCa patients and sIRGs, we analyzed the correlations of IRRSM and the clinical and demographic characteristics including age, gender, stage, T-stage, N-stage and M-stage. We found the expression levels of INHBA were enhanced in the advanced T-stages (Fig. 5A); the expression levels of INHBA and JAG2 decreased in the early N-stages (Fig. 5B); the expression levels of JAG2 were elevated in advanced M-stages (Fig. 5C). The area under curve (AUC) of receiver operating characteristic (ROC) curve represented the accuracy of the model. The AUC value of IRRSM was 0.661, indicating the accuracy of IRRSM is satisfied. However, the AUC of age, gender, stage, T-stage, N-stage and M-stage were 0.551, 0.483, 0.788, 0.661, 0.701 and 0.744 respectively, which suggested predicting prognosis based on some clinical and demographic characteristics was not always accurate (Fig. 5D).

### The clinical application of the IRRSM

In order to determine whether IRRSM could be used as an independent prognostic factor, we conducted the independent risk factor analysis. The results showed that stage, T-stage, N-stage, M-stage and IRRSM were remarkably correlated with OS in univariate analysis (*P* < 0.05). However, in the multivariate analysis, only age and IRRSM were significantly correlated with OS, which suggested the IRRSM could be regarded as an independent prognostic factor (Table 4). To further investigate the applications of IRRSM, we established a nomogram of CCa patients utilizing multivariate COX analysis of 3 sIRGs in the IRRSM (Fig. 5E). We normalized the points of each patient to a distribution ranging from 0 to 100, thus we could evaluate the 1-year, 3-year and 5-year survival probability of CCa patients by drawing a vertical line between the total points axis and each prognosis axis. The nomogram would provide a novel method for clinical workers to evaluate the prognoses of CCa patients.

**Table 4:** Univariate and multivariate analysis of CCa

Variables	Univariate analysis				Multivariate analysis			P value
	HR	HR 95% low	HR 95% high	P value	HR	HR 95% low	HR 95% high	
Age	1.020054	0.998836	1.041722	0.064108	1.033164	1.010137	1.056715	0.004553
Gender	1.230625	0.711617	2.010508	0.407324	1.002063	0.604320	1.661585	0.993626
Stage	2.605814	1.947303	3.487011	1.16E−10	1.506127	0.663146	3.420689	0.327813
T-stage	3.199674	1.942852	5.269528	4.89E−06	1.616453	0.885217	2.951728	0.118024
M-stage	5.821289	3.512203	9.648475	8.32E−12	2.132929	0.696311	6.533551	0.184760
N-stage	2.186720	1.639655	2.916310	1.01e−07	1.241346	0.750865	2.052219	0.399296
Risk score	1.251956	1.149711	1.363295	2.34E−07	1.181951	1.081014	1.292314	0.000242

*HR* Hazard ratio

### The immune status analysis of the high-risk and low-risk groups

**Fig. 6:**
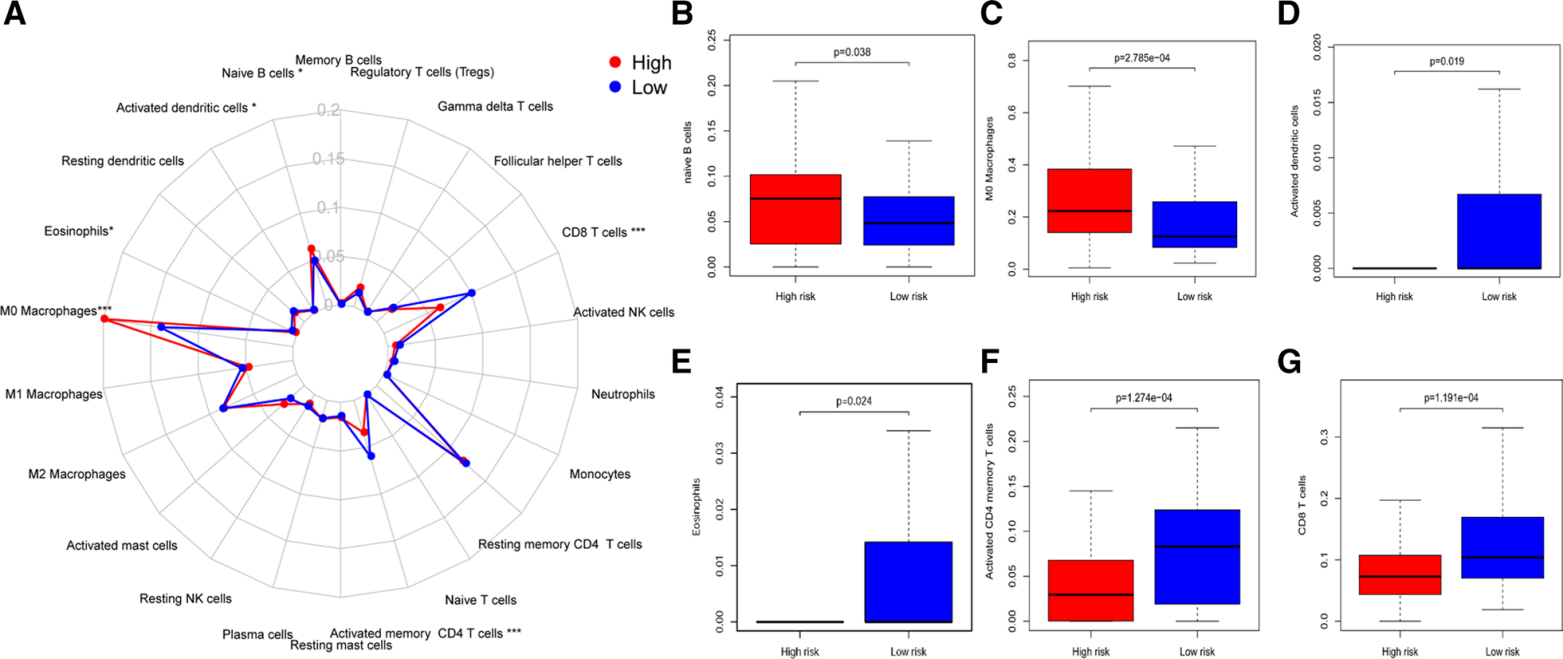
Relationships between the IRRSM and immune cells. The relationships between IRRSM and immune cells infiltration: radar plot (**A**); naive B cells (**B**); M0 macrophages (**C**); activated dendritic cells (**D**); eosinophils (**E**); activated memory CD4 T cells (**F**); CD8 T cells (**G**)

The PCA was employed to detect the different distribution patterns between the high-risk group and the low-risk group based on the immune gene expression sets and the genome-wide expression sets. In the genome-wide expression sets, we didn’t detect the significant separation between the high-risk group and the low-risk group (Fig. 5F). Next, we use GSEA enrichment analysis to analyze the pathway enrichment of these three sIRGs, and predicted that these three genes were all related to intestinal immune-related pathways. (Additional file 2: Figure S2). However, based on the immune risk gene sets, the high-risk group and the low-risk group were obviously separated into two parts (Fig. 5G). In order to further investigate whether the IRRSM accurately reflect the tumor immune microenvironment, we analyzed the relationships between the IRRSM and immune cells infiltration (Fig. 6A). We found naive B cells (Fig. 6B) and M0 macrophages (Fig. 6C) displayed the positive relevance with risk score, while activated dendritic cells (Fig. 6D), eosinophils (Fig. 6E), activated CD4 memory T cells (Fig. 6F) and CD8T cells (Fig. 6G) showed the opposite results. These results motivated us to further discover the underlying functions and mechanisms in future studies.

### INHBA and JAG2 were overexpressed in patients with CRC, especially with advanced T-stages

**Fig. 7:**
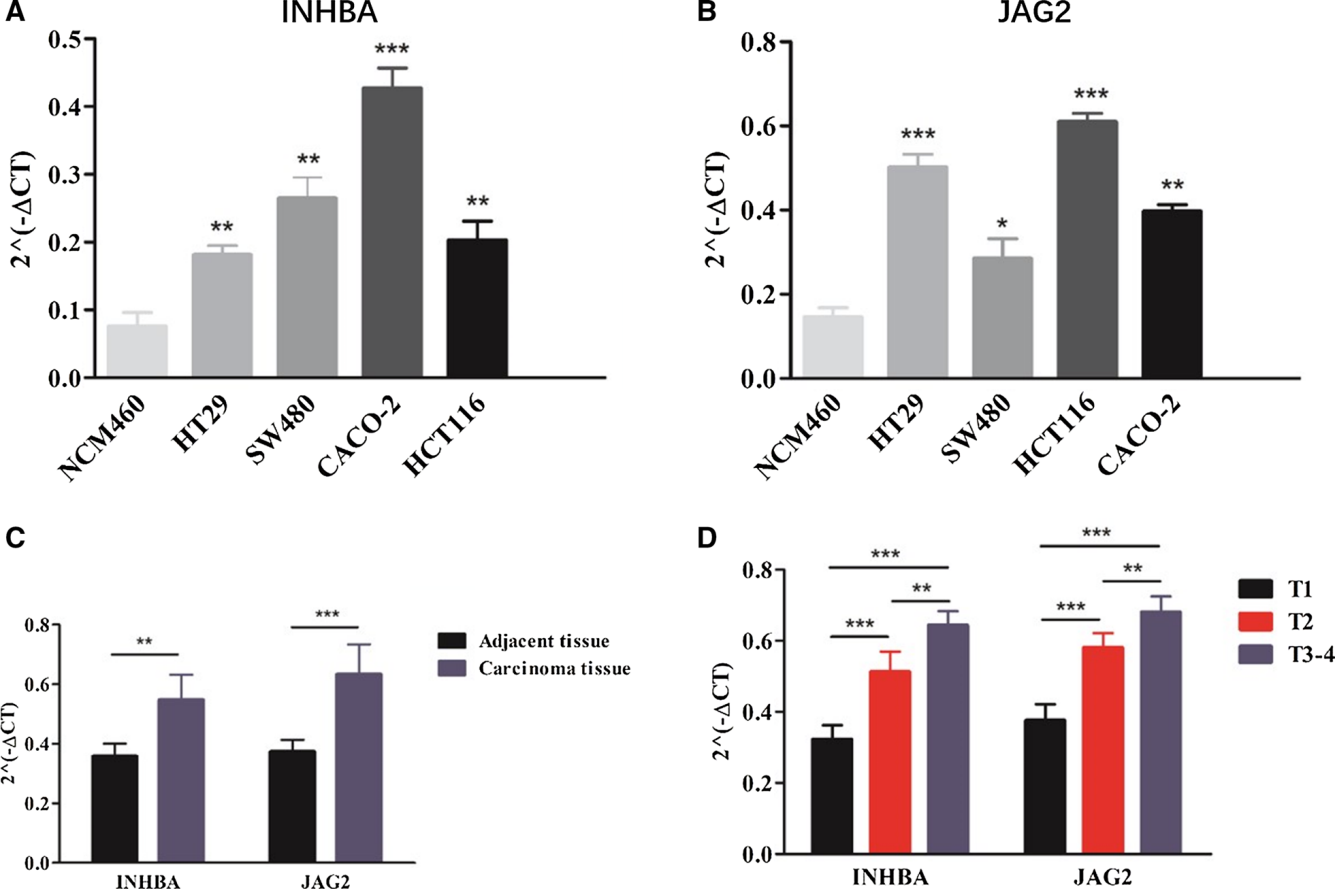
The expression levels of INHBA and JAG2 in patients with CCa and its correlation with T-stages. The results of RT-qPCR of INHBA’ (**A**) and JAG2’ (**B**) expression levels in colonic cancer cell lines. ***, ** and * represent the remarkable difference compared with NCM460 (*P* < 0.001, *P* < 0.01 and *P* < 0.05) respectively. The expression levels of INHBA and JAG2 in carcinoma tissues and adjacent tissues (**C**). *** and ** represent the significant difference compared with adjacent tissues (*P* < 0.001 and *P* < 0.01) respectively. The expression levels of INHBA and JAG2 in CCa tissues with various T-stages. *** and ** represent the remarkable difference compared with another group (*P* < 0.001 and *P* < 0.01) respectively

To further validated the predicting value of the IRRSM, we detect the expression levels of two sIRGs of the IRRSM (INHBA and JAG2) in vivo and in vitro, and their correlations with certain clinicopathologic features in vivo. As illustrated in the Fig. 7, the expression levels of INHBA (Fig. 7A) and JAG2 (Fig. 7B) in colonic cancer cell lines (CACO-2, HT29, SW480 and HCT116) were significantly higher than that in colonic epithelial cell line (NCM460). Besides, we further explored the correlations of INHBA and JAG2 with T-stage in vivo. As illustrated in Fig. 7C, INHBA and JAG2 both expressed more in carcinoma tissues than that in adjacent tissues. Additionally, the gradually higher expression levels of INHBA and JAG2 were detected in CRC tissues with more advanced T-stages (Fig. 7D).

## Discussion

Although surgery and postoperative adjuvant therapies to some extent improve the survival of CRC patients, the response rate and treatment efficiency remain unsatisfactory because of drug resistance and poor sensitivity and tolerance [4]. Therefore, exploring more individualized therapies based on the tumor features and foreseeable treatment outcomes will provide the probability for improving therapeutic efficiency and prolonging survival.

With the more thorough understanding of the significance of immune activities in the tumorigenesis, progression and prognosis of tumors, emerging immunotherapies have been occupying a high-profile position in the field of cancer treatment [19]. A growing body of immunotherapy drugs including pembrolizumab, ipilimumab and nivolumab have been approved and acquired rewarding effects [20, 21]. Consequently, discovering more promising and sensitive immune-related biomarkers such as IRGs attracted increasing attentions.

In recent years, many studies illustrated that tumor microenvironment (TME) played an important role in the prognoses of patients [22]. The significant value of TME in breast cancer has been reported by Soysal SD [23]. The researchers also found the effects of tumor immunity on prognoses and clinical decision-making. Jerome Galon highlighted the prominent efficacy of the immune contexture and immunoscore in cancer prognosis and therapy [24]. Jerome Galon's article highlighted the relationships between various immune cells and the prognosis of CCa patients. In the present study, we identified and selected three IRGs, not immune cells, to establish a risk model assessing the prognosis of CCa patients. The scoring method and system is somewhat different from that of Jerome Galon. The prognostic model of this 3 IRGs remains to be further verified by follow-up prospective studies and amounts of basic experiments. Besides, the intertwined histories of tumor immunology and tumor Evolution have also been brough forward and increasingly emphasized [25]. So, researchers began to turn their eyes on the roles of gene expression levels in tumor immunity. A great number of studies reported that some IRGs have the potential to predict and evaluate the effects of immunotherapies [26]. Therefore, the prediction value of tumors prognoses by IRGs become one of the hot research fields. The prognoses of 16 unique IRGs (HSPA1A, HSPA1B, HSPA5, MICB, PSMC3, TAP2, KIAA0368, RBP1, APOD, VDR, PPP3R1, IL11RA, LGR4, NRP1, PLCG1, GZMB) in gastric cancer was reported by Jiang [27]. Yangyang She reported that 27 IRGs could predict the prognosis of head and neck squamous cell carcinoma [28]. Although the characteristics and significances of IRGs in tumor invasion, metastasis, progression and immune-related response have been well confirmed in some cancers, the genome-wide and complete analysis of the mechanisms of CRC by IRGs were still confusing and needed to be fully explored [29].

In our study, we analyzed 667 down-regulated and 883 up-regulated differentially expressed genes in 437 patients with CRC, and combined with 311 immune genes screened from M13664 and M19817 of Molecular Signatures Database. We finally selected 29 IRGs correlated with CCa. We further explored the interactions and potential pathway mechanisms of 29 IRGs. Subsequently, we investigated the relationships between the expression levels of IRGs and the prognoses of patients with CCa by univariate COX analysis, and 6 IRGs were significantly correlated with OS. Based on multivariate Cox analysis, 3 sIRGs were further screened to establish an IRRSM, and CCa patients were divided into high-risk group and low-risk group. The survival possibility in the low-risk group was significantly higher than that in the high-risk group. We further verified the predictive value of IRRSM by univariate COX analysis and multivariate COX analysis. The results showed that IRRSM could be regarded as an independent prognostic factor to evaluate the prognoses of patients with CCa. We found that the expression levels of INHBA were increased in the more advanced stages and T-stages; the expression levels of INHBA and JAG2 were increased in advanced N-stages; the expression levels of JAG2 were decreased in early M-stages. We also constructed a nomogram to enable the clinical workers to predict the 1-, 3- and 5-years survival probability of CCa patients. As a result of the infiltration of immune cells components and the low purity of tumor are positively correlated with tumor malignant progression, enhanced immunophenotype and poor prognoses [30, 31]. Therefore, we used PCA to explore the different distribution patterns of low-risk group and high-risk group on the basis of genome-wide expression sets and immune risk gene expression sets. When PCA was performed based on genome-wide expression sets, the immune status of these groups did not show significant separation. However, according to the immune risk gene expression sets, the low-risk group and the high-risk group tend to be separated into two parts. Then we analyzed the relationships between immune infiltration and IRRSM. The naïve B cells and M0 macrophages were higher in the high-risk group. While activated dendritic cells, eosinophils, activated CD4 memory T cells and CD8 T Cells were higher in the low-risk group. Therefore, IRRSM was closely related to the immune status of patients with CCa. These results suggested that IRRSM could help us identify the high-risk patients from the patients who have the same clinical or molecular characteristics. Therefore, clinical workers could achieve individualized and appropriate treatment strategies for CCa patients.

In order to verify the reliability and accuracy of our results, we identified the expression levels of INHBA and JAG2 in CCa tumor tissues, adjacent tissues, human colonic epithelial cell line and colonic cancer cell lines. The expression levels of INHBA and JAG2 in colonic epithelial cell line were remarkably lower than that in colonic cancer cell lines. Moreover, the higher expression levels of INHBA were correlated with the tumor tissues of the more advanced T-stages.

Although we illuminated the value of IRRSM in predicting the prognoses in CCa patients and the expression levels of some sIRGs (JAG2 and INHBA) in IRRSM of CCa, there are still some limitations in our study. In the first place, we should combine our study with proteomics, metabonomics and immunology tests to achieve a deeper understanding of these sIRGs. Therefore, the practical value of sIRGs should to be fully clarified and extensively verified. Secondly, the other compositions in IRRSM should be detected in vivo and in vitro.

## Conclusion

In the present manuscript, we analyzed the roles of sIRGs in predicting and evaluating the clinical prognoses of CCa patients and verified the predictive value of some sIRGs. Our results provide a new perspective for immunotherapies and establish a reliable and accurate IRRSM to predict the prognoses of CCa patients.

## Supplementary Information

**Additional file 1: Figure S1.** The workflow of the experiment.

**Additional file 2: Figure S2.** The GSEA analysis of sIRGs. The GSEA analysis of CCL19 (A) JAG2 (B) and INHBA (C).

## Data Availability

Authors can provide all of datasets analyzed during the study on reasonable request.

## Abbreviations

CCa: Colon cancer
CRC: Colorectal cancer
IME: Immune microenvironment
IRGs: Immune-related genes
IRRSM: Immune-related risk score model
OS: Overall survival
sIRGs: IRGs with significant clinical outcomes
TCGA: The Cancer Genome Atlas
TME: Tumor microenvironment
