# Supplementary figures and images for: An immune-related model based on INHBA, JAG2 and CCL19 to predict the prognoses of colon cancer patients
Source: Cancer Cell Int. 2021 Jun 8;21:299. doi: 10.1186/s12935-021-02000-z (PMC8186192; doi:10.1186/s12935-021-02000-z)

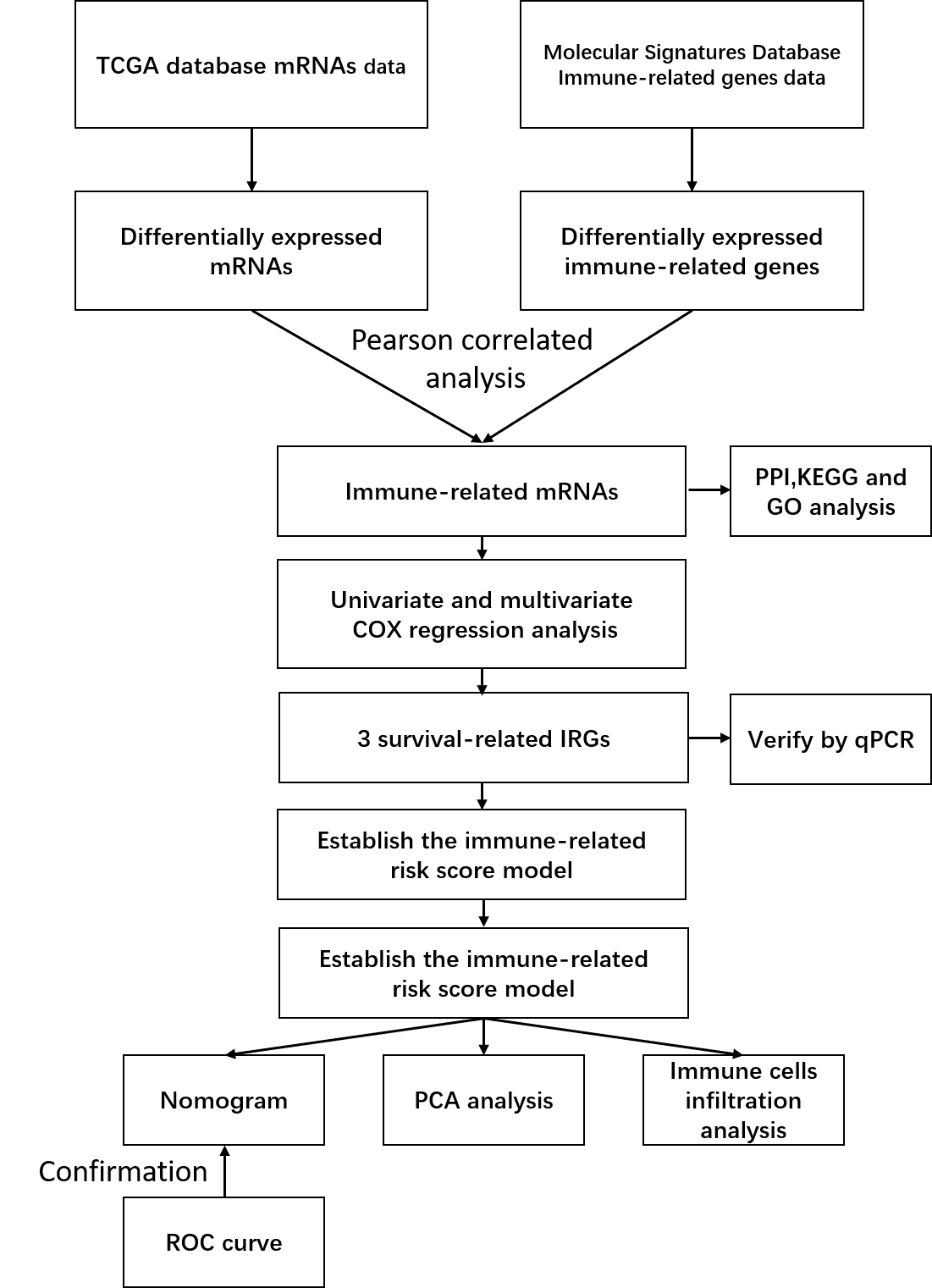

Supplement: Supplementary file 1 — Additional file 1: Figure S1. The workflow of the experiment. [file 12935_2021_2000_MOESM1_ESM.tif]

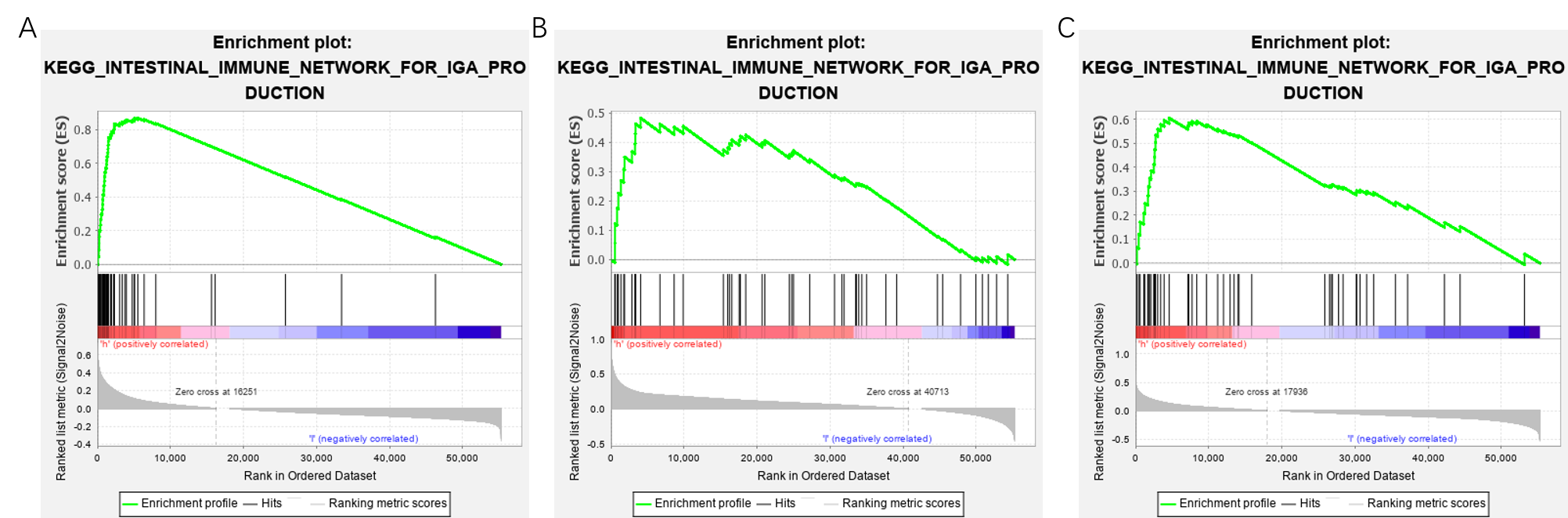

Supplement: Supplementary file 2 — Additional file 2: Figure S2. The GSEA analysis of sIRGs. The GSEA analysis of CCL19 (A) JAG2 (B) and INHBA (C). [file 12935_2021_2000_MOESM2_ESM.tif]
